# Physical activity and sedentary behavior among students and teachers in disadvantaged secondary schools: a cross-sectional study from northern Hungary

**DOI:** 10.1016/j.pmedr.2026.103564

**Published:** 2026-07-08

**Authors:** Erika Beregi, József Bognár

**Affiliations:** a University of Miskolc, Faculty of Health, Miskolc, Hungary; b Eszterházy Károly Catholic University, Sport and Health Science Research Group, Eger, Hungary; c Eszterházy Károly Catholic University, Institute of Sport Science, Eger, Hungary

**Keywords:** Physical activity, Sedentary behavior, Adolescents, Teachers, Disadvantaged region, School health

## Abstract

**Objective:** To describe physical activity and sedentary behavior among students and teachers in disadvantaged secondary schools in Northern Hungary and to examine differences by gender, grade level, teacher age, and respondent group. **Methods:** A cross-sectional online survey was conducted in spring 2022 in 11 state-maintained secondary schools in Northern Hungary. The sample included 732 students in grades 10 to 12 and 102 full-time teachers. Measures were adapted from the Health Behavior in School-aged Children questionnaire and the International Physical Activity Questionnaire Short Form. Group differences were tested using *t-*tests, analysis of variance, linear regression, and multivariate analysis of variance. **Results:** Students reported an average of 3.44 days per week with at least 60 min of continuous physical activity, while 23.6% reported no moderate physical activity. Boys were significantly more active than girls. Among teachers, 28.0% reported no vigorous activity. Teachers' estimates of students' activity were lower than students' self-reports. **Conclusions:** Insufficient vigorous activity and prolonged sedentary time were common in both groups. Whole-school interventions should address students' and teachers' behaviors in disadvantaged school contexts.

## Highlights

• Students and teachers reported low vigorous physical activity.
• Students reported more total physical activity than teachers.
• Prolonged sitting was common among students and teachers.
• Teacher estimates were lower than student self-reports.
• Whole-school programs should address students and teachers.

## 1 Introduction

Sedentary behavior is associated with cardiovascular disease risk, and the negative health effects increase as sitting time and inactivity accumulate (Katzmarzyk et al., 2019). Regular physical activity contributes to the prevention of cardiovascular disease, diabetes, cancer, musculoskeletal problems, and mental health problems (Pedersen and Saltin, 2015; Warburton and Bredin, 2017; González and Fuentes, 2017; Schuch and Vancampfort, 2021). In adolescence, physical activity is particularly important for development, well-being, and physical and mental health (Janssen and LeBlanc, 2010; Marques et al., 2019; Poitras et al., 2016). Activity intensity, duration, and frequency are central to interpreting these benefits, and metabolic equivalents are commonly used to distinguish light, moderate, and vigorous activity (Haskell et al., 2007).

Adolescent physical activity is shaped by socioeconomic status, family background, school environment, community conditions, and organized sport participation (Sollerhed et al., 2021). Despite international recommendations, inactivity remains common among adolescents in Central and Eastern Europe (Kantanista et al., 2021). Screen time may further reduce physical activity, sleep quality, and well-being (Borfe et al., 2025; Ganesamoorthy et al., 2024).

Teachers are also relevant from a preventive health perspective because schools are both educational settings and workplaces. Teaching is associated with musculoskeletal disorders, psychological strain, and burnout risk (Erick and Smith, 2011), while physical activity has been linked to better teacher mental health (Aperribai et al., 2020). Schools are therefore important both as educational settings and as workplaces where organizational conditions may influence health behavior (Wilf Miron et al., 2022). Kroupis et al. (2019) also showed that the availability of school sports facilities was associated with physical education teachers' job satisfaction and burnout.

In disadvantaged regions, physical activity is shaped not only by individual motivation but also by the opportunities available in the local environment. Sociocultural and socioeconomic constraints may limit access to safe recreational spaces, organized sport, and supportive environments for physical activity (Nikitara et al., 2021). Evidence from disadvantaged Hungarian settlements has also shown low levels of sport participation, suggesting that regional disadvantage may restrict opportunities for active leisure time (Lukács and Lenténé Puskás, 2022).

This study is guided by socioecological models and the social determinants of health framework, which view physical activity and sedentary behavior as outcomes of interacting individual, social, institutional, community, and structural factors (Sallis et al., 2006; World Health Organization, 2008). In this study, gender, grade level, self-rated fitness, physical activity, and sitting time represent individual-level characteristics, while teachers' own physical activity and their estimates of students' activity reflect social and institutional features of the school environment.

The social determinants of health framework emphasizes that disadvantage may restrict access to health-promoting resources and contribute to persistent inequalities (Marmot et al., 2008). In disadvantaged regions, limited access to safe recreational spaces, organized sport, transport opportunities, and supportive environments may shape students' physical activity through family resources, local infrastructure, school opportunities, and peer norms (Tremblay et al., 2016). Teachers' physical activity may also be influenced by workload, workplace culture, available facilities, and broader living conditions.

Teachers may contribute to health-promoting school environments through role modeling, daily expectations, and motivational support. From a self-determination theory perspective, teachers can support students' autonomy, competence, and relatedness through everyday instructional practices, thereby contributing to a supportive motivational climate (Ahmadi et al., 2023). Burnout among physical education teachers also shows that teacher well-being is a relevant public health concern within the school setting (Alsalhe et al., 2021). Therefore, examining students and teachers within the same disadvantaged school context can provide an integrated understanding of physical activity and sedentary behavior as shared public health challenges.

Self-report questionnaires remain feasible in large school-based physical activity studies, although their reliability and validity vary (Silsbury et al., 2015; Ács et al., 2021). Although adolescent physical activity has been widely studied, less attention has been given to teachers in disadvantaged regions and to comparisons between students and teachers within the same schools. Teachers may act as role models in school-based health promotion, yet their own physical activity may also be insufficient (Kwiecień-Jaguś et al., 2021). Comparing students and teachers within the same school context is important because teachers may shape health-related norms, motivational climate, and the implementation of whole-school health promotion.

The primary aim of this study was to describe physical activity patterns, sedentary behavior, and self-rated fitness among students and teachers in disadvantaged secondary schools in Northern Hungary. Secondary aims were to compare physical activity levels between students and teachers and to examine differences by gender, grade level, and teacher age group.

## 2 Methods

### 2.1 Study design and population

This cross-sectional study was conducted in spring 2022 in state-maintained secondary schools in Northern Hungary, one of the most disadvantaged regions of the country according to national development indicators. The study focused on general secondary schools in order to keep the educational setting comparable. Vocational and technical schools were not included.

Of the 22 eligible state-maintained general secondary schools in the region, 11 agreed to participate after administrative permissions had been obtained, giving a school-level participation rate of 50%. This participation rate may have introduced selection bias, as schools agreeing to participate may have differed from non-participating schools in their interest in health promotion, administrative capacity, or willingness to engage in research.

The target population included students in grades 10 to 12 and full-time teachers working in the participating schools. These grades represent late adolescence, when physical activity habits, sedentary behavior, and health-related routines become increasingly established.

The starting and analytical sample included 834 respondents, consisting of 732 students and 102 full-time teachers. No respondents were excluded from the analytical sample. Among students, 27.2% were in grade 10, 38.3% in grade 11, and 32.7% in grade 12. The mean age of students was 17.3 ± 1.2 years, and 64.9% were male. The mean age of teachers was 48.2 ± 11.4 years, and 62.4% were female. Teacher age was treated exploratorily, both as a grouped variable and as a continuous predictor in regression models. The most frequent teaching areas were languages, mathematics, vocational or specialist subjects, and physical education (Table 1)

**Table 1:** Distribution of sociodemographic characteristics among secondary school students and teachers in disadvantaged schools in Northern Hungary, spring 2022.

Characteristic	Students	Teachers
Age, mean ± SD	17.3 ± 1.2	48.2 ± 11.4
Gender	64.9% male, 35.1% female	62.4% female, 37.6% male
County seat city	14.8%	46.5%
Town	40.6%	35.6%
Village or farm	44.7%	17.8%

### 2.2 Measures and statistical analysis

Data were collected using an online questionnaire administered through Google Forms™. Students completed the survey during school hours in computer-equipped classrooms or during free time, while teachers completed it individually.

The questionnaire included sociodemographic items and physical activity and sedentary behavior items adapted from the Health Behavior in School-aged Children questionnaire and the International Physical Activity Questionnaire Short Form. These items assessed weekly physical activity, vigorous exercise, moderate physical activity, walking, sitting time, screen-based sedentary behaviors, and time spent preparing for lessons. The instruments have been used in international and Hungarian research (Beregi and Bognár, 2023; Craig et al., 2003; Inchley et al., 2020).

Physical activity outcomes were expressed as days per week, times per week, minutes per day, and total weekly metabolic equivalent minutes where applicable. Teachers also estimated students' physical activity and fitness, allowing comparison with student self-reports. Because the study used self-reported behavioral frequency and duration items rather than multi-item psychological scales, internal consistency estimates were not calculated for all variables.

Descriptive statistics were used to describe the sample and the variables of the study. Continuous variables are presented as means and standard deviations, and categorical variables are presented as percentages. Group differences were examined using independent-samples *t-*tests and one-way analysis of variance, as appropriate. Levene's test was used to assess the homogeneity of variances, and Welch's test was applied when variances were unequal.

Gender differences, grade-level differences, and differences between students and teachers were examined for selected physical activity and sedentary behavior indicators. Teacher age was treated exploratorily as both a grouped variable and a continuous predictor in linear regression models. A two-way multivariate analysis of variance examined the effects of gender and respondent group on selected physical activity indicators. Because Box's M test was significant, Pillai's Trace was used for multivariate interpretation.

Effect sizes were reported where appropriate. Cohen's d was used for selected independent-samples *t-*tests, and partial eta squared was used for analysis of variance and multivariate results. Given the exploratory nature of the study and the number of comparisons conducted, statistically significant findings were interpreted with attention to effect size and practical relevance. Statistical significance was set at *p* < 0.05. Analyses were conducted using IBM SPSS Statistics®, version 25.0.

### 2.3 Ethical considerations

Written informed consent was obtained from all participants before data collection. For students under 18 years of age, parental or guardian consent was also obtained. The study followed the Declaration of Helsinki and institutional guidelines for the protection, safety, and privacy of human participants. Ethical approval was granted by the Research Ethics Committee of Eszterházy Károly Catholic University (Approval ID: RK/1369/2022).

## 3 Results

### 3.1 Students' physical activity and sedentary behavior

Students reported at least 60 min of continuous physical activity on 3.44 ± 2.00 days during the seven days before data collection. For a usual week, the value was similar, at 3.49 ± 1.90 days. Vigorous physical activity outside school occurred 3.14 ± 1.71 times per week (Table 2).

**Table 2:** Descriptive statistics for physical activity and sedentary behavior based on the Health Behavior in School-aged Children questionnaire among secondary school students in disadvantaged schools in Northern Hungary, spring 2022.

Activity	Mean	SD	95% confidence interval
Vigorous exercise outside school, times per week	3.14	1.71	3.02, 3.27
Watching Television or video viewing, weekdays, minutes per day	153.19	133.27	143.46, 162.93
Watching Television or video viewing, weekends, minutes per day	228.28	117.29	219.71, 236.85
Playing electronic devices, weekdays, minutes per day	108.15	109.12	100.16, 116.14
Playing electronic devices, weekends, minutes per day	159.83	141.74	149.48, 170.18
Non-gaming electronic device use, weekdays, minutes per day	175.79	118.03	167.17, 184.43
Non-gaming electronic device use, weekends, minutes per day	202.41	126.28	193.18, 211.66
Preparing for lessons, weekdays, minutes per day	84.95	75.65	79.41, 90.49
Preparing for lessons, weekends, minutes per day	97.67	92.51	90.92, 104.43

Note. SD = standard deviation.

Screen-based activities were higher on weekends. Television or video viewing increased from 153.20 ± 133.27 min per day on school days to 228.28 ± 117.29 min on weekends. Playing electronic devices increased from 108.15 ± 109.12 to 159.83 ± 141.74 min per day, and non-gaming electronic device use increased from 175.80 ± 118.03 to 202.42 ± 126.28 min per day.

According to International Physical Activity Questionnaire Short Form, 23.6% of students reported no moderate physical activity during the previous week, while 20.1% reported approximately 30 min of moderate-intensity activity per day. Nearly half, 49.0%, reported walking for at least 10 min on all seven days. The largest proportion, 45.7%, reported sitting for more than seven hours per day (Table 3).

**Table 3:** Descriptive statistics for physical activity and sitting time based on the International Physical Activity Questionnaire Short Form among secondary school students in disadvantaged schools in Northern Hungary, spring 2022.

Variable	Mean	SD	95% confidence interval
Vigorous physical activity, days per week	2.34	1.99	2.20, 2.49
Vigorous physical activity duration, minutes per day	73.35	71.46	67.42, 79.28
Moderate physical activity, days per week	2.20	1.91	2.06, 2.34
Moderate physical activity duration, minutes per day	55.96	65.39	50.55, 61.38
Walking, days per week	5.46	1.89	5.32, 5.60
Walking duration, minutes per day	52.88	69.26	47.79, 57.98
Sitting time, minutes per day	339.04	165.72	326.92, 351.17

Note. SD = standard deviation.

Across grade levels, no significant differences were found in the main physical activity indicators. Differences were found only for weekend television viewing (F = 3.35, *p* = 0.03), weekday lesson preparation (F = 4.65, *p* = 0.01), and weekend lesson preparation (F = 3.29, p = 0.03). Tenth-grade students watched the most television, while twelfth-grade students spent the most time preparing for lessons. Grade level showed a weak negative association with days of vigorous physical activity (β = −0.07, *p* = 0.04). Boys reported more days with at least 60 min of physical activity (*t* = 5.31, *p* < 0.01), more extracurricular physical activity (*t* = 4.72, p < 0.01), and longer activity duration (*t* = 4.50, p < 0.01) than girls.

### 3.2 Teachers' assessments of students' physical activity

Teachers rated students' overall fitness as good (2.84 ± 0.79), while stamina was rated lower (2.06 ± 0.71). They estimated that students achieved at least 60 min of physical activity on 1.88 ± 1.49 days in the previous week, lower than students' self-reported values (Table 4). No significant differences were found by teacher age group (*t* = 1.16, *p* = 0.24). Male teachers estimated school-day television and video viewing to be higher than female teachers did (*t* = 2.68, *p* = 0.01).

**Table 4:** Comparison of secondary school students' self-reported physical activity and teachers' evaluations of student physical activity in disadvantaged schools in Northern Hungary, spring 2022.

Indicator	Students, mean ± SD	Teachers' estimate, mean ± SD	t value	*p* value	Cohen's *d*
Days with at least 60 min physical activity /week	3.44 ± 2.00	1.88 ± 1.49	8.22	<0.01	0.57
Vigorous physical activity, times per week	3.14 ± 1.71	2.39 ± 1.60	5.54	<0.01	0.59
Overall fitness rating	Not applicable	2.84 ± 0.79	Not applicable	Not applicable	Not applicable

Note. *p*-values were obtained from independent-samples t-tests. SD = standard deviation.

### 3.3 Teachers' physical activity and sedentary behavior

Among teachers, 28.0% reported no vigorous physical activity and 17.8% reported no moderate physical activity during the previous week. Teachers engaged in vigorous activity on 1.95 ± 1.80 days per week and moderate activity on 2.45 ± 2.11 days per week. Mean sitting time was 215.10 ± 121.63 min per day (Table 5).

**Table 5:** Descriptive statistics for physical activity and sitting time based on the International Physical Activity Questionnaire Short Form among teachers in disadvantaged secondary schools in Northern Hungary, spring 2022.

Variable	Mean	SD	95% confidence interval
Vigorous physical activity, days per week	1.95	1.80	1.59, 2.31
Vigorous physical activity duration, minutes per day	65.61	61.63	52.63, 78.60
Moderate physical activity, days per week	2.45	2.11	2.04, 2.87
Moderate physical activity duration, minutes per day	60.36	69.20	46.27, 74.47
Walking, days per week	4.42	2.28	3.97, 4.88
Walking duration, minutes per day	37.44	25.81	32.27, 42.63
Sitting time, minutes per day	215.09	121.63	191.09, 239.11

Note. SD = standard deviation.

Exploratory linear regression analyses showed no significant associations between teacher age and vigorous activity, moderate activity, walking, or sitting time. The strongest but non-significant associations were found for moderate activity days, F(1, 97) = 3.70, *p* = 0.06, R^2^ = 0.04, moderate activity duration, F(1, 91) = 3.57, p = 0.06, R^2^ = 0.04, and walking days, F(1, 97) = 3.31, *p* = 0.07, R^2^ = 0.03.

### 3.4 Comparison of students and teachers

Total weekly physical activity was 3134.50 MET-minutes per week among students and 2424.68 among teachers (Table 6). Vigorous physical activity contributed the largest share in both groups. Students reported significantly higher vigorous activity and walking than teachers, whereas moderate activity did not differ significantly.

**Table 6:** Comparison of total weekly metabolic equivalent minutes for physical activity between secondary school students and teachers in disadvantaged schools in Northern Hungary, spring 2022.

Activity type	Students, metabolic equivalent min/week	Teachers, metabolic equivalent min/week	t value	p value	Cohen's *d*
Vigorous physical activity	1593.33	1151.68	2.11	0.03	0.22
Moderate physical activity	596.97	720.99	−0.80	0.42	−0.08
Walking	944.20	552.01	5.76	<0.01	0.61
Total	3134.50	2424.68	–	–	–

During the previous week, 24.1% of students and 28.0% of teachers reported no vigorous physical activity. Students rated their own fitness higher than teachers rated students' fitness (*t* = 9.64, *p* < 0.01). Students also reported more walking (*t* = 4.22, p < 0.01) and sitting time (*t* = 9.24, p < 0.01) than teachers.

A two-way multivariate analysis of variance examined the effects of gender and respondent group (student vs. teacher) on selected physical activity indicators. Box's M test was significant (M = 278.43, p < 0.01); therefore, Pillai's Trace was used. Multivariate main effects were significant for gender (F = 3.19, *p* = 0.01, partial η^2^ = 0.03) and respondent group (F = 4.84, p < 0.01, partial η^2^ = 0.05), as was the gender × respondent group interaction (F = 2.49, *p* = 0.02, partial η^2^ = 0.03). Follow-up analyses showed that male participants reported more days of moderate physical activity (F = 9.58, p = 0.01, partial η^2^ = 0.01), and students reported more walking days than teachers (F = 15.18, p < 0.01, partial η^2^ = 0.02) with small effect sizes.

## 4 Discussion

This study examined physical activity among secondary school students and teachers in one of Hungary's most disadvantaged regions. Insufficient vigorous physical activity and prolonged sitting were common in both groups. These findings support socioecological and social determinants perspectives, which emphasize that health behaviors are shaped by individual, institutional, and structural conditions (Sallis et al., 2006; Marmot et al., 2008).

The results are consistent with evidence that many adolescents and adults do not achieve sufficient physical activity, particularly in Central and Eastern Europe (Tremblay et al., 2016; Kantanista et al., 2021). Relatively high total Metabolic equivalent values occurred alongside long sitting time, suggesting that everyday movement may coexist with prolonged sedentary behavior, a pattern relevant for chronic disease prevention (Katzmarzyk et al., 2019). This pattern should not be interpreted as contradictory, as adolescents may engage in structured or vigorous physical activity while also accumulating prolonged sedentary time through screen use, studying, and seated leisure activities.

Students reported higher total activity than teachers, mainly through vigorous activity and walking. However, both groups included participants with no vigorous activity during the previous week, suggesting that movement opportunities during the school day may be relevant for both students and teachers.

Boys reported more physical activity than girls, consistent with international Health Behavior in School-Aged Children Questionnaire findings (Inchley et al., 2020). The MANOVA results also supported the role of gender and respondent group, but the effect sizes were small, suggesting that the practical magnitude of these differences should be interpreted cautiously. The gender gap may reflect norms, opportunities, and barriers rather than motivation alone, highlighting the need for gender-sensitive physical activity programs (Wiltshire et al., 2019). In disadvantaged Hungarian regions, gender differences may be shaped by unequal access to organized sport, family resources, and school-based activity opportunities. Therefore, these differences should be interpreted not only as individual behavioral patterns, but also as outcomes influenced by social and environmental constraints.

Teachers' estimates of students' physical activity were lower than students' self-reports. This discrepancy should be interpreted cautiously because student self-reports may be affected by recall bias, social desirability bias, and overestimation, while teachers' estimates may reflect a narrower school-day observational context. Therefore, the difference may indicate measurement bias in either respondent group rather than genuine teacher underestimation.

Health Behavior In School-Aged Children Questionnaire items and International Physical Activity Questionnaire Short Form indicators should be interpreted as complementary. The Health Behavior In School-Aged Children Questionnaire items describe context-specific routines, whereas International Physical Activity Questionnaire Short Form focuses on accumulated physical activity by intensity and duration (Dowd et al., 2018; Silsbury et al., 2015).

The relatively low level of vigorous activity among teachers is important because teachers are employees, health educators, and potential role models. Limited teacher engagement in physical activity may reduce the credibility and sustainability of whole-school health promotion (Wilf Miron et al., 2022). Teacher age did not significantly explain teachers' activity or sitting-time patterns, suggesting that inactivity among teachers should be interpreted as a broader workplace and health-promotion issue rather than an age-specific pattern.

Overall, the findings suggest that health promotion in disadvantaged secondary schools should use a multilevel approach. School leaders can support movement opportunities during the school day, curriculum planning can include short activity breaks and active learning strategies, and teacher training can strengthen role-modeling capacity and confidence in health promotion. Partnerships with community-based physical activity programs may further expand opportunities beyond the classroom (Sallis et al., 2006).

This study has limitations. The cross-sectional design does not allow causal conclusions. Physical activity and sedentary behavior were self-reported and may be affected by recall bias, social desirability bias, and possible overestimation, particularly among adolescents. Although half of the eligible schools participated, selection bias may have occurred because participating schools may have differed from non-participating schools. The findings should therefore be interpreted as context-specific and should not be generalized beyond disadvantaged secondary schools in Northern Hungary without caution. The study did not include objective activity measures or detailed school-level variables, such as school size, physical education provision, sports facilities, extracurricular opportunities, or institutional socioeconomic indicators. Future research using objective measures and multilevel modeling could clarify how institutional conditions shape physical activity patterns.

## 5 Conclusions

Insufficient physical activity and prolonged sedentary time are shared challenges among students and teachers in disadvantaged secondary schools. Students reported higher total physical activity than teachers, but both groups included participants with no vigorous activity during the previous week. Differences between students' self-reports and teachers' evaluations highlight the need for contextual interpretation of subjective measures. Future studies should combine objective tools with validated self-report instruments. School-based interventions addressing both student and teacher needs may help reduce inactivity and improve health equity in socioeconomically vulnerable educational environments.

## Availability of data and materials

The data are available from the corresponding author upon reasonable request, subject to ethical and privacy restrictions.

## CRediT authorship contribution statement

**Erika Beregi:** Writing – review & editing, Writing – original draft, Methodology, Data curation, Conceptualization. **József Bognár:** Writing – review & editing, Writing – original draft, Supervision, Methodology, Conceptualization.

## Consent for publication

The manuscript has been approved by all authors.

## Ethics approval and consent to participate

This study was conducted in accordance with the principles of the Declaration of Helsinki. Institutional ethical approval was granted by the Research Ethics Committee of Eszterházy Károly Catholic University (Approval ID: RK/1369/2022).

Written informed consent was provided by all participating teachers and students. For students under 18 years of age, written informed consent was also obtained from parents or legal guardians.

## Declaration of generative AI and AI-assisted technologies in the writing process

During the preparation of this work, the authors used ChatGPT, a large language model developed by OpenAI, to support language editing and improve clarity and readability. After using this tool, the authors reviewed and edited the content as needed and take full responsibility for the content of the published article.

ORCID IDs.

Erika Beregi: 0009-0008-3163-3704.

József Bognár: 0000-0002-5982-7833.

## Funding

This research received no specific grant from any funding agency in the public, commercial, or not-for-profit sectors.

## Declaration of competing interest

The authors declare that they have no known competing financial interests or personal relationships that could have appeared to influence the work reported in this paper.

## Data Availability

Data will be made available on request.
